# Using a patient-centred composite endpoint in a secondary analysis of the Control of Hypertension in Pregnancy Study (CHIPS) Trial

**DOI:** 10.1186/s13063-023-07118-1

**Published:** 2023-02-07

**Authors:** Rebecca K. Metcalfe, Mark Harrison, Joel Singer, Mary Lewisch, Terry Lee, Peter von Dadelszen, Laura A. Magee, Nick Bansback, the CHIPS Study Group

**Affiliations:** 1 Centre for Health Evaluation & Outcome Sciences, St. Paul’s Hospital, 1081 Burrard Street, Vancouver, British Columbia V6Z 1Y6 Canada; 2 School of Population and Public Health, University of British Columbia, 2206 East Mall, Vancouver, British Columbia V6T 1Z3 Canada; 3 Faculty of Pharmaceutical Sciences, University of British Columbia, 2405 Wesbrook Mall, Vancouver, British Columbia V6T 1Z3 Canada; 4 Patient Partner, BC Support Unit, 420-1367 West Broadway, Vancouver, British Columbia V6H 4A7 Canada; 5 Department of Women and Children’s Health, School of Life Course Sciences, Faculty of Medicine, King’s College London, Becket House, 1 Lambeth Palace Road, Room BH.05.11, London, SE1 7EU UK; 6 BC SUPPORT Unit, 420-1367 West Broadway, Vancouver, British Columbia V6H 4A7 Canada

**Keywords:** Patient-centred, Randomized controlled trial, Composite endpoints, Pregnancy hypertension, Perinatal

## Abstract

**Background:**

Clinical trials commonly use multiple endpoints to measure the impact of an intervention. While this improves the comprehensiveness of outcomes, it can make trial results difficult to interpret. We examined the impact of integrating patient weights into a composite endpoint on the interpretation of Control of Hypertension in Pregnancy Study (CHIPS) Trial results.

**Methods:**

Outcome weights were extracted from a previous patient preferences study in pregnancy hypertension (*N* = 183 women) which identified (i) seven outcomes most important to women (taking medication, severe hypertension, pre-eclampsia, blood transfusion, Caesarean, delivery < 34 weeks, and baby born smaller-than-expected) and (ii) three preference subgroups: (1) ‘equal prioritizers’, 62%; (2) ‘early delivery avoiders’, 23%; and (3) ‘medication minimizers’, 14%.

Outcome weights from the preference subgroups were integrated with CHIPS data for the seven outcomes identified in the preference study. A weighted composite score was derived for each participant by multiplying the preference weight for each outcome by the binary outcome if it occurred. Analyses considered equal weights and those from the preference subgroups. The mean composite scores were compared between trial arms (*t*-tests).

**Results:**

Composite scores were similar between trial arms with the use of equal weights or those of subgroup (1) (95% confidence intervals [CIs]: − 0.03, 0.02; *p* > 0.50 for each). ‘Tight’ control was superior when using subgroup (2) weights (95% CIs: 0.002, 0.07; *p* = 0.03), and ‘less-tight’ control was superior when using subgroup (3) weights (95% CIs: − 0.11, − 0.04; *p* < 0.01).

**Conclusions:**

Evidence-based recommendations for ‘tight’ control are consistent with most women’s preferences, but for a sixth of women, ‘less-tight’ control is more preference consistent. Depending on patient preferences, a single trial may support different interventions. Future trials should specify component weights to improve interpretation.

**Trial registration:**

ClinicalTrials.gov NCT01192412

**Supplementary Information:**

The online version contains supplementary material available at 10.1186/s13063-023-07118-1.

## Introduction

Clinical trials in cardiovascular medicine routinely use primary, secondary, and other endpoints to capture the breadth of an intervention’s effects. However, this can make the interpretation of trial results challenging, as an intervention’s effects can vary by outcome, including benefits and harms [1]. Composite endpoints are often used to overcome these challenges, particularly in pregnancy, “ … to circumvent a contrived prioritization of one-half of the mother–infant pair and acknowledge the interconnectedness of mothers and babies at the time of childbirth.” [2] Composites are typically dichotomous, and treat outcomes as equal, which may not be the case. To apply trial results in practice, clinicians and patients must consider which endpoints are important to them and to what degree [3].

The international Control of Hypertension in Pregnancy Study (CHIPS; ClinicalTrials.gov NCT01192412) [4] randomized controlled trial (RCT) compared ‘less-tight’ with ‘tight’ control of blood pressure (BP) for management of chronic or gestational hypertension; women who progressed to preeclampsia remained in their allocated group. ‘Less-tight’ control aimed to minimize antihypertensive therapy (target diastolic BP of 100 mmHg), while ‘tight’ control aimed to normalize BP (target diastolic BP of 85 mmHg). While ‘tight’ (vs. ‘less-tight’) control did not change the incidence of the primary foetal/newborn and secondary maternal composite outcomes (with equally valued components) [4], ‘tight’ control has been recommended by many guidelines based on a decrease in severe maternal hypertension and some preeclampsia-related complications [5–8]. These findings were recently replicated in a separate trial [9]. However, recommendations did not integrate women’s preferences or concerns, like taking medications during pregnancy [10].

In a secondary analysis of CHIPS Trial data, we explored whether weighting outcomes to reflect patient preferences would change the interpretation of trial results.

## Methods

We integrated pregnant women’s preferences for the management of pregnancy hypertension [11] with individual event data from the CHIPS Trial [4].

Outcome data from the 981 women enrolled in CHIPS were included (Table 1). The inclusion criteria were as follows: 14^+0^–33^+6^ weeks’ gestation, nonproteinuric chronic or gestational hypertension, office diastolic BP of 90–105 mmHg (or 85–105 mmHg if the women were taking antihypertensive medication), and a live foetus [4]. On average, participants were ≈ 34 years of age and enrolled at ≈ 24 weeks. Most (75%) women had chronic hypertension. Roughly half were taking antihypertensives.

**Table 1:** CHIPS trial event rates of the seven outcomes women prioritized, overall and by trial arm^a^

Outcome (presence of)	Outcome data from CHIPS trial [4]			Weights from preference study [11]			
	Overall (***N*** = 981)	Trial arm		Equal weights	Subgroups based on patient preference weights^**b**^		
		‘Less-tight’ control (***N*** = 493)	‘Tight' control (***N*** = 488)		(1) (***N*** = 114)	(2) (***N*** = 44)	(3) (***N*** = 25)
Prescribed antihypertensives	837 (85.3%)	379 (76.9%)^c^	458 (93.9%)^c^	14%	14%	2%	58%
Severe hypertension	334 (34.0%)	200 (40.6%)^c^	134 (27.5%)^c^	14%	11%	20%	20%
Pre-eclampsia	464 (47.3%)	241 (48.9%)	223 (45.7%)	14%	15%	16%	10%
Blood transfusion	24 (2.4%)	16 (3.2%)	8 (1.6%)	14%	20%	2%	0%
Caesarean	481 (49.0%)	231 (47.0%)	250 (51.4%)	14%	13%	2%	4%
Delivery < 34 weeks	138 (14.1%)	77 (15.7%)	61 (12.6%)	14%	18%	42%	2%
BW < 10th percentile	175 (17.8%)	79 (16.1%)	96 (19.8%)	14%	8%	16%	5%

*BW* birthweight

^a^Of the 987 women randomized in CHIPS, outcomes were available for 981 following 6 withdrawals and losses to follow-up, with the exception of antihypertensive medication for which data were available for 986 women [4]

^b^Subgroups (1) ‘equal prioritizers’, (2) ‘early delivery avoiders’, and (3) ‘medication minimizers’ [11]

^c^The difference between the groups was statistically significant at the *p* < 0.001 level

Preferences were obtained from a separate study [11], in which 183 pregnant women in Canada prioritized CHIPS Trial outcomes, including the primary perinatal (pregnancy loss and/or neonatal care unit admission > 48 h) and secondary maternal outcomes (serious maternal complications). In semi-structured focus groups and individual interviews, participants identified five maternal and two foetal/newborn outcomes as important and sufficiently different between treatment arms to influence their preferred BP control (Table 1) [4]. Preference subgroup weights were derived from a best-worst scaling task (BWS). In this task, participants were shown a series of choice sets each comprising four of the seven prioritized outcomes and asked to select the outcome that was most important to them to avoid and the outcome that was least important to them to avoid [12]. As the BWS used a balanced-incomplete block design, all outcomes were presented the same number of times and compared to all other attributes once. BWS analyses used Latent Gold 5.1 [13]. Conditional logit models of BWS responses quantified the relative value of each prioritized outcome (where each outcome’s relative importance was expressed as a proportion, and all components summed to 100%) [11]. Latent class analysis identified three preference subgroups and their respective weights (Table 1): (1) *equal prioritizers* (62%) who placed fairly equal weight on each outcome, (2) *early delivery avoiders* (23%) who prioritized avoiding delivery before 34 weeks (weight of 42%), and (3) *medication minimizers* (14%) who prioritized avoiding antihypertensive medication (weight of 58%).

We considered equal weights (as assumed in conventional analysis) and the three preference subgroup weights. For each approach, a composite score was derived for each CHIPS trial participant by multiplying the patient preference weight for each outcome by the binary outcome of its occurrence [14]. Thus, higher composite scores indicated worse outcomes (more highly weighted events occurred). The mean composite scores between interventions were compared using *t*-tests with an a priori *p*-value set at *<* 0.05. Analyses were conducted using RStudio [15].

A threshold analysis for preference subgroups that supported ‘less-tight’ over ‘tight’ control was conducted to determine the extent to which preferences would need to shift to yield a finding congruent with current clinical guidance. The threshold analysis systematically reduced the weight assigned to the most highly weighted composite component and distributed the removed weight across the six other components proportionately to the weight assigned in the preference profile. The proportional weight for a given outcome was calculated as the weight assigned to that outcome divided by the sum of the weights assigned to all of the outcomes except the highest weighted outcome. For example, using subgroup (3) weights, the weight assigned to severe hypertension would increase by 0.047 which is equal to 0.01 (the weight reduction) multiplied by the weight assigned to severe hypertension (0.20) and divided by 1 minus the subgroup (3) weight assigned to minimizing antihypertensive medication—the highest weighted outcome (1 − 0.58 = 0.42). These redistributed weights were calculated for each one-point reduction in the highest weighted composite component. The primary analysis (*t*-test) was then repeated for each set of redistributed weights.

This study was reviewed and approved by the Behavioural Research Ethics Board (H17-01194) at the University of British Columbia.

## Results

Table 2 shows that using equal weights in the composite score produced no difference in score between treatment arms; the significantly higher frequency of antihypertensive medication use in ‘tight’ control was offset by the significantly higher frequency of severe hypertension in ‘less-tight’ control. Similar results were found using subgroup (1) weights (*equal prioritizers*).

**Table 2:** Mean weighted composite outcome score^a^ by blood pressure control, and *t* scores for each analysis

	‘Less-tight’ control	‘Tight’ control	95% CI	*t*-test results		
			Lower	Upper	*t*	*p*
**Equal weights**	0.35	0.36	− 0.03	0.02	− 0.43	0.67
**Subgroup (1):** ‘equal prioritizers’	0.33	0.34	− 0.03	0.02	− 0.34	0.73
**Subgroup (2):** ‘early delivery avoiders’	0.28	0.24	0.002	0.07	2.12	0.03
**Subgroup (3):** ‘medication minimizers’	0.61	0.68	− 0.11^b^	− 0.04^b^	− 4.14	< 0.01

^a^Lower scores indicate fewer highly weighted events occurred

^b^Favours ‘less-tight’ control

Using subgroup (2) weights (*early delivery avoiders*), the apparently lower rate of early delivery (and significantly lower incidence of severe hypertension) in the ‘tight’ control arm resulted in a lower (better) composite outcome score for ‘tight’ (vs ‘less-tight’) control. The use of significantly more antihypertensive therapy in ‘less-tight’ control contributed little given the low weighting of this outcome (Table 2).

Using subgroup (3) weights (*medication minimizers*), the significantly lower frequency of antihypertensive medication use in ‘less-tight’ (vs. ‘tight’) control, combined with a high weighting (58%) resulted in a significantly lower (better) composite outcome score, despite significantly more severe hypertension (20% weight) (Table 2).

**Fig. 1:**
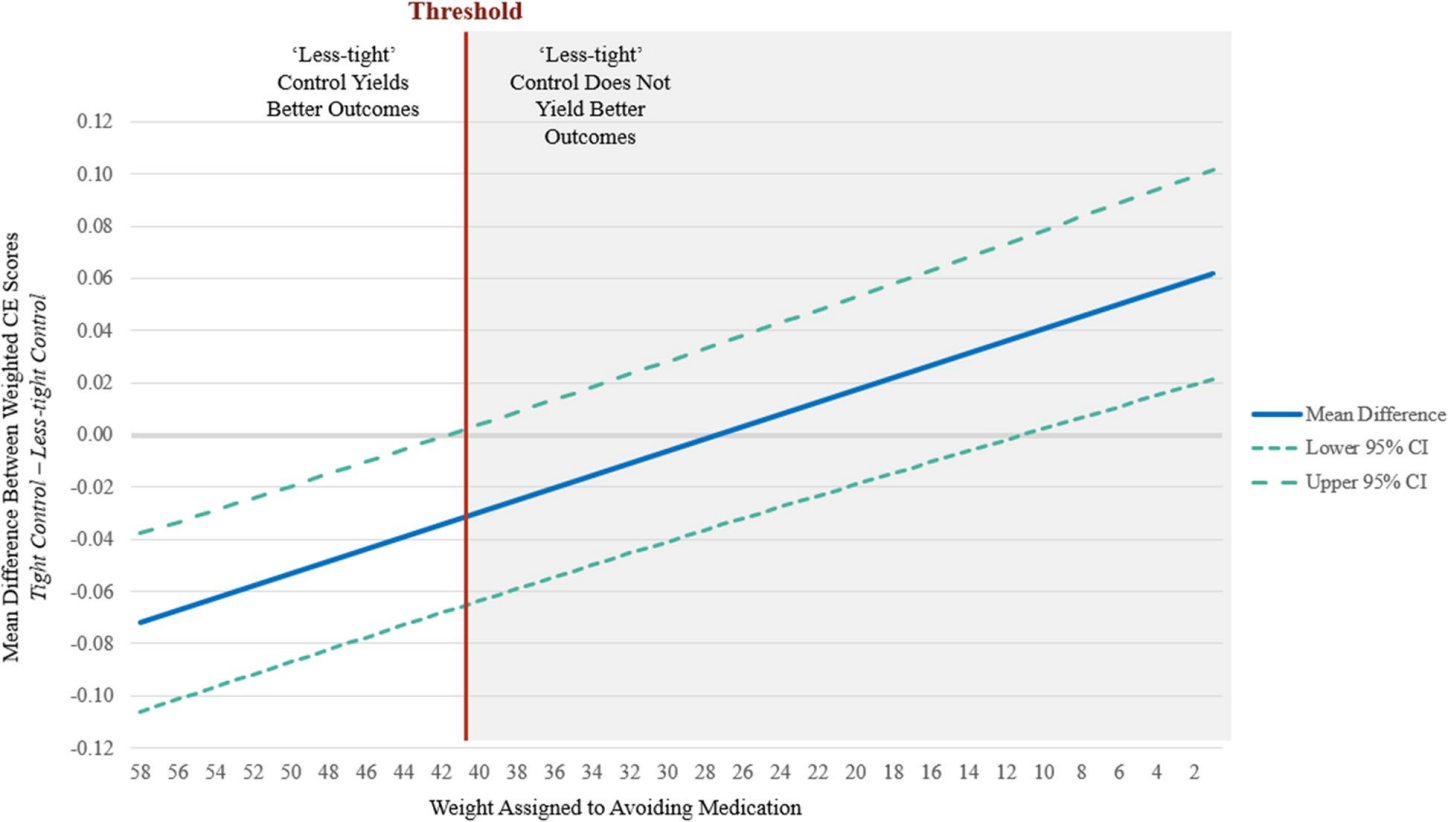
Threshold analysis results for subgroup (3) *medication minimizers*

The threshold analysis conducted for subgroup (3) showed that once the weight applied to avoiding antihypertensive medication was reduced to 0.41 (from 0.58), ‘less-tight’ control was no longer the preferred treatment (Fig. 1).

## Discussion

This re-analysis of CHIPS trial outcomes incorporated patient views and demonstrated that integrating patient preferences for outcomes and their associated weights into trial analyses is feasible and can identify different management approaches based on the results of a single trial. Our findings suggest that while almost two-thirds of women prioritize adverse outcomes equally, as assumed in the primary CHIPS analyses, about one-quarter prioritize very preterm birth that clearly favours ‘tight’ control. A distinct minority prioritize minimizing antihypertensive medication above other adverse outcomes, making ‘less-tight’ control the most value-congruent BP management for them.

Recent clinical practice guidelines have recommended ‘tight’ control of pregnancy hypertension [5–8], based on the findings of a significant reduction in the development of severe hypertension and some preeclampsia-related complications, without an increase in perinatal risk, from CHIPS [4] and other RCTs [16]. Our findings suggest that ‘tight’ control is appropriate for the vast majority (≈ 85%) of pregnant women.

While integrating preferences into composites has been considered in cardiology [3, 14] and other fields [17], this is the first study to integrate patient weights with individual event data from a high-quality RCT in pregnancy. Our findings show that specifying outcome weights may change the interpretation of trial results when applied to individual women. Importantly, our methods are easily adapted to other trial and non-trial approaches and can be used with other statistical methods that accommodate confounders and covariates (e.g. linear regression; ANCOVA).

Limitations of our work include the use of preference weights that reflect women’s values in Canada; despite its multiethnic population, values may differ elsewhere. Preferences were identified after CHIPS was completed; consequently, different composite components may have been identified a priori. However, CHIPS evaluated the standard obstetric outcomes that cover most of the subsequently published relevant core outcome set. These results are statistically significant at the group level, but clinical significance likely depends on individual preferences. Additionally, our method of preference elicitation may have been too cognitively burdensome for some participants. BWS was chosen because it can provide cardinal importance values on an additive scale. As a result, weights can be directly compared to one another and the magnitude of the difference in the importance of outcomes is known. Alternative methods of analysis which use ranks, rather than weights, to incorporate the importance of composite components were considered [18–20]. These approaches have advantages in that ranks may be easier to ascertain and more intuitive to use, but they also pose some challenges. For example, ranking approaches that compare intervention and control participant outcomes in order of composite component importance often stop at the first difference in component outcomes (e.g., win ratio [18]). These approaches risk excluding information on lower ranked components that are still important to patients. Other methods that use all components (e.g., O’Brien’s global rank method [20]) can lack specificity on how to rank components (e.g., rank all outcomes [20] or rank hierarchically [21]) and on how to address ties between participants. While a weighting approach seemed most appropriate for our analysis, there are potential benefits of a rank-based analysis in other contexts which should be considered in future composite analyses. Finally, our approach presents challenges for statistical power (e.g., power calculations), although these come with the benefit of improved interpretability.

## Conclusions

This study illustrates that integrating patient values into trial analyses can change the interpretation of trial results for clinical decision-making. Future trials with composite or multiple outcomes should seek patient preference weights to improve the interpretation of trial results and support patient-centred care.

## Supplementary Information

**Additional file 1: Table S1.** CHIPS Study Group.

## Data Availability

The datasets used and/or analysed during the current study are not publicly posted as participant consent was not obtained for the open distribution of data. However, data are available from the corresponding author upon reasonable request. Aggregate data are available as part of the Supplementary Appendix of the initial publication of the CHIPS trial results (10.1056/NEJMoa1404595).

## Abbreviations

ANCOVA: Analysis of covariance
BP: Blood pressure
BWS: Best-worst scaling
CHIPS: Control of Hypertension in Pregnancy Study
RCT: Randomized controlled trial
BW: Birth weight
