# Supplementary material for: Using a patient-centred composite endpoint in a secondary analysis of the Control of Hypertension in Pregnancy Study (CHIPS) Trial
Source: Trials. 2023 Feb 7;24:99. doi: 10.1186/s13063-023-07118-1 (PMC9906819; doi:10.1186/s13063-023-07118-1)
Supplement: Supplementary file 1 — Additional file 1: Table S1. CHIPS Study Group. [file 13063_2023_7118_MOESM1_ESM.docx]

**Table S1: CHIPS Study Group**

| **Steering Committee**: Laura A. Magee (Chair), Elizabeth Asztalos, Amiram Gafni, Andrée Gruslin, Michael Helewa, Eileen Hutton, Alexander Logan, Jennifer Menzies, Jean-Marie Moutquin, Kellie Murphy, Evelyne Rey, Sue Ross, Johanna Sanchez, Joel Singer, Peter von Dadelszen |
| --- |
| **Working Group**: Laura A. Magee (Chair), Elizabeth Asztalos, Peter von Dadelszen, Trinh Hoac, Joanne Kirton, Jennifer Menzies, Sue Ross, Johanna Sanchez, Katherine Trigiani, Ainy Zahid |
| **Adjudication Committee**: Laura A. Magee (Chair), Elizabeth Asztalos, Kellie Murphy, Evelyne Rey, Peter von Dadelszen |
| **Data Safety Monitoring Board**: Michael B. Bracken (Chair), Patricia Crowley, Lelia Duley, Richard Ehrenkranz, Kevin Thorpe |
| **Data programmers and analysts**: Sunny Chan, Michael Shi, Shelley Yu |
| **Collaborators**: The number of women recruited in each country and centre is specified in brackets. |
| **ARGENTINA** (36 women): |
| **Hospital LC Lagomaggiore**, Mendoza (16): Raquel de Lourdes Martin, Maria Florencia Bassi, Mirta Clara Caruso, Valeria Lagunas, Fernando Vera |
| **Hospital Avellaneda**, Tucuman (10): Maria Mohedano de Duhalde, Alicia Beatriz Roque, Patricia Roldan, Esteban Marcos Duhalde, Viviana Dip |
| **Hospital JR Vidal**, Corrientes (8): Jesus Daniel Aguirre, Elba Mirta Alicia Morales, Griselda Itati Abreo, Teresa De Sagastizabal, Carolina Gomez, Nadia Rizzi |
| **Hospital JM Cullen**, Santa Fe (2): Carlos Arias, Ricardo Antonio Bruno |
| **AUSTRALIA** (85 women): |
| **Ipswich Hospital**, Ipswich (36): Kassam Mahomed, Alison Drew, Ann Green, Jane Hoare |
| **Women's and Children's Hospital**, Adelaide (18): Bill Hague, Suzette Coat, Caroline Crowther, Peter Muller , Sophie Trenowden |
| **King Edward Memorial Hospital**, Subiaco (17): Barry Walters, Claire Parker, Dorothy Graham, Craig Pennell, Eileen Sung |
| **Campbelltown Hospital**, Penrith South (8): Angela Makris, Gaksoo Lee, Charlene Thornton, Annemarie Hennessy |
| **Liverpool Hospital**, Penrith South (5): Angela Makris, Gaksoo Lee, Charlene Thornton, Annemarie Hennessy |
| **St John of God Hospital**, Subiaco (1): Louise Farrell, Claire Parker, Eileen Sung, Barry Walters |
| **BRAZIL** (19 women): |
| **Maternidade de Vila Nova Cachoeirinha**, Sao Paulo (7): Nelson Sass, Henri Korkes, Dayana Couto Ferreira |
| **Hospital Universitario Antonio Pedro**, Niteroi (6): Renato Augusto Moreira de Sa, Monique Schmidt Marques Abreu |
| **Maternidade Escola da UFRJ**, Rio de Janeiro (4): Rita Guerios Bornia, Nancy Ribeiro da Silva, Fernanda Freitas Oliveira Cardoso |
| **Hospital Sao Lucas - PUCRS**, Porto Alegre (2): Caio Coelho Marques, Jorge Hornos, Ricardo Leal Davdt, Letícia Germany Paula, Pedro Luis Zanella |
| **CANADA** (233 women): |
| **British Columbia Women's Hospital and Health Centre**, Vancouver (32): Laura A. Magee, Peter von Dadelszen, Gabrielle Inglis, Ruth Dillon, Ashley Docherty, Anna Hutfield |
| **Jim Pattison Outpatient Care and Surgery Centre**, Surrey (26): Keith Still, Sayrin Lalji, Tamara Van Tent, Chris Hotz, Tracy Messmer |
| **St Michael's Hospital**, Toronto (22): Joel G. Ray, Howard Berger, Leanne De Souza, Andrea Lausman, Tatiana Freire-Lizama, Kate Besel |
| **Foothills Medical Centre**, Calgary (21): Paul Gibson, Greta Ellsworth, Leslie Miller, T. Lee-Ann Hawkins |
| **Sunnybrook Health Sciences Centre**, Toronto (19): Michelle Hladunewich, Anna Rogowsky, Dini Hui, Virginia Collins |
| **IWK Health Centre**, Halifax (19): Isabelle Delisle, Cora Fanning |
| **Royal Alexandra Hospital**, Edmonton (16): Nestor Demianczuk, Rshmi Khurana, Winnie Sia, Catherine Marnoch, Carmen Young, Cheryl Lux |
| **CHU Sainte-Justine**, Montreal (15): Evelyne Rey, Sophie Perreault, Valerie Tremblay |
| **CHUS Fleurimont**, Sherbrooke (13): Jean-Marie Moutquin, Sophie Desindes, Anne-Marie Côté, Veronique Dagenais |
| **Ottawa Hospital Civic Division**, Ottawa (13): Andrée Gruslin, Heather Clark, Elaine O'Shea, Ruth Rennicks White |
| **Mount Sinai Hospital**, Toronto (8): Shital Gandhi, Mary-Jean Martin, Cheryl Brush, Gareth Seaward |
| **Royal University Hospital**, Saskatoon (6): Jill Newstead-Angel, Judy Brandt, Jocelyne Martel, Kristine Mytopher, Elise Buschau |
| **Ottawa Hospital General Division**, Ottawa (5): Andree Gruslin, Erin Keely, Patti Waddell, Ruth Rennicks White, Svetlana Shachkina, Alan Karovitch |
| **St Paul's Hospital**, Vancouver (5): Robert Anderson, Nicole Koenig, Theresa Yong |
| **Toronto East General Hospital**, Toronto (5): Marie Vasiliou, Peri Johnson, Beth Allan |
| **London Health Sciences Centre**, London (4): Renato Natale, Laura Kennedy |
| **Royal Victoria Hospital**, Montreal (2): Lucie Opatrny, Lorraine Lavigne |
| **Regina General Hospital**, Regina (1): George Carson, Sheila Kelly |
| **Women’s Health Centre**, St John's (1): Joan Crane, Donna Hutchens |
| **CHILE** (57 women): |
| **Hospital Dr Sotero del Rio**, Puente Alto (45): Juan Pedro Kusanovic, Christian Figueroa, Karla Silva Neculman, Juan Andres Ortiz, Paula Vargas |
| **Hospital Base Osorno**, Osorno (12): Pedro Ferrand, Jorge Carrillo |
| **COLOMBIA** (36 women): |
| **Corporacion Comfenalco Valle - Universidad Libre** (20), **Clinica Versalles** (11), **Clinica Materno Infantil Farallones** (5), Cali: Rodrigo Cifuentes Borrero, Dahiana Marcela Gallo, Luisa Fernanda Moreno |
| **ESTONIA** (19 women): |
| **Tartu University Hospital - Women's Clinic**, Tartu (19): Fred Kirss, Kristiina Rull, Anne Kirss |
| **HUNGARY** (5 women): |
| **University of Debrecen**, Debrecen (5): Tamas Major, Andrea Fodor, Tunde Bartha |
| **ISRAEL** (12 women): |
| **Hillel Yaffe Medical Center**, Hadera (6): Mordechai Hallak, Nardin Aslih, Saja Anabousi-Murra, Ester Pri-Or |
| **Ma'ayney Hayeshua Medical Center**, Bnei Brak (3): Linda Harel, Sima Siev |
| **Nazareth Hospital (EMMS)**, Nazareth (3): Marwan Hakim, Christina Simona Khoury, Najla Hamati |
| **JORDAN** (13 women): |
| **Islamic Hospital**, Amman (13): Mazen El-Zibdeh, Lama Yousef |
| **NEW ZEALAND** (17 women): |
| **Christchurch Women's Hospital**, Christchurch (16): Ruth Hughes, Di Leishman, Barbra Pullar |
| **Waitemata Health-North Shore Hospital**, Auckland (1): Matthew Farrant |
| **POLAND** (21 women): |
| **Medical University of Gdansk**, Gdansk (9): Malgorzata Swiatkowska-Freund, Krzysztof Preis, Anette Aleksandra Traczyk-Los, Anna Partyka, Joanna Preis-Orlikowska, Mariusz Lukaszuk |
| **Polish Mothers Memorial Hospital**, Lodz (9): Grzegorz Krasomski, Michael Krekora, Anna Kedzierska-Markowicz, Katarzyna Zych-Krekora |
| **University School of Medical Sciences**, Poznan (3): Grzegorz H. Breborowicz, Anna Dera-Szymanowska |
| **THE NETHERLANDS** (96 women): |
| **Academic Medical Center**, Amsterdam (28): Wessel Ganzevoort, Jannet Bakker, Joost Akkermans, Anouk Pels |
| **OLVG**, Amsterdam (13): Eline van den Akker, Sabine Logtenberg |
| **UMCU**, Utrecht (10): Steven Koenen, Maartje de Reus, David Borman, Martijn A. Oudijk |
| **VU Medical Center**, Amsterdam (9): Annemiek Bolte, Viki Verfaille, Bart Graaf |
| **Maxima Medical Centre**, Veldhoven (8): Martina Porath, Corine Verhoeven, Ben Willem Mol |
| **UMCG**, Groningen (6): Maureen T.M. Franssen, Lida Ulkeman, Ineke Hamming, Jose H.M. Keurentjes, Ina van der Wal |
| **Isala Klinieken Zwolle**, Zwolle (5): S.W.A. Nij Bijvank, A.A. Lutjes |
| **Tergooiziekenhuizen**, Hilversum (5): Henricus Visser, Jannet Bakker |
| **MUMC Maastricht**, Maastricht (4): Hubertina Catharina Johanna Scheepers |
| **St Antonius Ziekenhuis**, Nieuwegein (3): Erik van Beek, David Borman, Coby van Dam, Kathy van den Berg-Swart |
| **Kennemer Gasthuis Haarlem**, Haarlem (2): Paula Pernet, Birgit van der Goes |
| **Diakonessen Ziekenhuis**, Utrecht (1): Nico Schuitemaker |
| **Flevo ziekenhuis**, Almere (1): Gunilla Kleiverda, Marcel van Alphen, Ageeth Rosman |
| **Jeroen Bosch Hospital**, 's-Hertogenbosch (1): Ingrid Gaugler-Senden, Marieke Linders |
| **UNITED KINGDOM** (268 women): |
| **Guy's & St Thomas' Hospital**, London (38): Catherine Nelson-Piercy, Annette Briley, May Ching Soh, Kate Harding, Hayley Tarft |
| **New Cross Hospital**, Wolverhampton (31): David Churchill, Katherine Cheshire, Julia Icke, Mausumi Ghosh |
| **Nottingham City Hospital**, Nottingham (30): James Thornton, Yvonne Toomassi, Karen Barker, Joanne Fisher, Nicky Grace, Amanda Green, Joanne Gower , Anna Molnar, Shobhana Parameshwaran, Andrew Simm |
| **Queen's Medical Centre**, Nottingham (22): James Thornton, George Bugg, Yvette Davis, Ruta Desphande, Yvette Gunn, Mohammed Houda, Anna Molnar, Nia Jones |
| **Royal Victoria Infirmary**, Newcastle upon Tyne (22): Jason Waugh, Carly Allan, Gareth Waring |
| **Liverpool Women's Hospital**, Liverpool (16): Steve A. Walkinshaw , Angela Pascall, Mark Clement-Jones, Michelle Dower, Gillian Houghton, Heather Longworth, Tej Purewal |
| **Bradford Royal Infirmary**, Bradford (13): Derek Tuffnell, Diane Farrar, Jennifer Syson, Gillian Butterfield, Vicky Jones, Rebecca Palethorpe, Tracey Germaine |
| **Leicester Royal Infirmary**, Leicester (12): Marwan Habiba, Debbie Lee |
| **Wexham Park Hospital**, Slough (12): Olufemi Eniola, Lynne Blake, Jane Khan |
| **City Hospitals Sunderland NHS Foundation Trust**, Sunderland (10): Helen M. Cameron, Kim Hinshaw, Amanda Bargh, Eileen Walton |
| **South Warwickshire NHS Trust**, Warwick (9): Olanrewaju Sorinola, Anna Guy, Zoe D'Souza, Rhiannon Gabriel, Jo Williams |
| **Derriford Hospital**, Plymouth (8): Ross Welch, Heidi Hollands |
| **York Hospital**, York (8): Olujimi Jibodu, Sara Collier, Pauline Tottie, Claire Oxby, James Dwyer |
| **Singleton Hospital**, Swansea (7): Franz Majoko, Helen Goldring, Sharon Jones |
| **Chesterfield Royal Hospital**, Chesterfield (6): **Janet Cresswell,** Louise Underwood**,** Mary Kelly-Baxter**,** Rebecca Robinson |
| **Sheffield Teaching Hospitals NHS Foundation Trust**, Sheffield (6): Dilly Anumba, Anne Chamberlain, Clare Pye |
| **St Mary's Hospital**, Manchester (6): **Clare Tower,** Sue Woods**,** Lisa Horrocks, Fiona Prichard, Lynsey Moorhead, Sarah Lee, Louise Stephens , Cara Taylor, Suzanne Thomas, Melissa Whitworth, Jenny Myers |
| **Birmingham Women's Hospital**, Birmingham (5): Ellen Knox, Katie Freitas, Mark Kilby, Amanda Cotterill |
| **Lancashire Teaching Hospitals NHS Foundation Trust**, Lancashire (3): Khalil Abdo, Katrina Rigby, Julie Butler, Fiona Crosfill, Sean Hughes, Sanjeev Prashar, Fatimah Soydemir |
| **The Royal Derby Hospital**, Derby (3): Janet Ashworth, Lorraine Mycock, Jill Smith |
| **Basildon & Thurrock University Hospital**, Basildon (1): **Amaju Ikomi,** Kerry Goodsell, Jean Byrne**,** Maxwell Masuku**,** Alice Pilcher |
| **USA** (70 women): |
| **Cooper University Hospital**, Camden (13): Meena Khandelwal, Gunda Simpkins, Michelle Iavicoli, Yon Sook Kim, Richard Fischer, Robin Perry |
| **Medical University of South Carolina**, Charleston (11): Eugene Y. Chang, Tamara D. Saunders, Betty W. Oswald, Kristin D. Zaks |
| **Beth Israel Deaconess**, Boston (8): Sarosh Rana, Dawn McCullough |
| **Yale-New Haven Hospital**, New Haven (8): Anna Sfakianaki, Cheryl Danton, Erin Kustan, Luisa Coraluzzi |
| **Norton Hospital Downtown** (7), **Norton Suburban Hospital** (2), Louisville: Helen How, Christina Waldon |
| **East Carolina University**, Greenville (6): Jeffrey Livingston, Sherry Jackson, Lisa Greene |
| **Meriter Hospital**, Madison (6): Dinesh Shah |
| **Oregon Health & Science University**, Portland (5): Jorge E. Tolosa, Monica Rincon, Leonardo Pereira, Amy E. Lawrence, Janice E. Snyder |
| **University of North Carolina**, Chapel Hill (4): D. Michael Armstrong, Teresa Blue, Austin Hester, Kathryn Salisbury |
